# Recent progress in the treatment of non-systemic juvenile idiopathic arthritis

**DOI:** 10.12703/r/10-23

**Published:** 2021-02-26

**Authors:** John M Bridges, Elizabeth D Mellins, Randy Q Cron

**Affiliations:** 1 Children’s of Alabama/University of Alabama at Birmingham, Birmingham, Alabama, USA; 2 Department of Pediatrics, Program in Immunology, Stanford University, Stanford, California, USA

**Keywords:** Juvenile idiopathic arthritis, uveitis, temporomandibular joint, biologic, DMARD

## Abstract

Juvenile idiopathic arthritis (JIA) is a chronic inflammatory disease affecting the joints and other organs that occurs in 1 in 1,000 children in the United States. Given the various categories of JIA, interpretation of the literature can be difficult. In this review, new developments in understanding non-systemic JIA and its treatment will be covered. Recent advances in the journey toward personalized treatment in JIA will be highlighted, including a review of currently available biologic modifiers. Uveitis and the temporomandibular joint will be discussed as particularly challenging treatment issues. Recent guideline updates and literature-guided treatment decisions will be reviewed.

## Introduction

Juvenile idiopathic arthritis (JIA) is a chronic inflammatory disease affecting the joints and other organs that occurs in 1 in 1,000 children in the United States^1^. Long-term complications of under-treated JIA include joint deformity, reduced quality of life, and significant disability^2,3^. The most widely used JIA classification, put forth by the International League of Associations for Rheumatology (ILAR), divides JIA into seven different groups based upon the number of joints affected and other accompanying features (see Table 1)^4^. With this many categories, clinical interpretation of the research literature can be difficult. While most categories of JIA share similarities in disease presentation and pathophysiology, systemic JIA, psoriatic arthritis, and enthesitis-related arthritis are separate disease entities^4^. In this review, new developments in understanding non-systemic JIA and its treatment will be covered. We will discuss updates on understanding personalized diagnosis and management of JIA, current biologic treatment options, and recent guidelines and treatment decision research.

**Table 1.** International League of Associations for Rheumatology classification criteria for juvenile idiopathic arthritis.

Category	Inclusion criteria	Exclusions
	Arthritis	
Systemic	Fever at least 2 weeks; daily for at least 3 days One or more of evanescent erythematous rash, lymphadenopathy, hepatomegaly, splenomegaly, or serositis	a,b,c,d
Oligoarthritis	Arthritis in 1–4 joints for the first 6 months	a,b,c,d,e
Polyarthritis (RF–)	Arthritis affecting 5 or more joints during the first 6 months of disease A test for RF (if performed) is negative	a,b,c,d,e
Polyarthritis (RF+)	Arthritis affecting 5 or more joints during the first 6 months of disease 2 or more tests for RF at least 3 months apart during the first 6 months of disease are positive	a,b,c,d,e
Psoriatic	Arthritis and psoriasis; or Arthritis accompanied by two of the following: dactylitis; nail pitting or onycholysis; or psoriasis in a FDR	b,c,d,e
ERA	Arthritis and enthesitis; or Arthritis or enthesitis accompanied by at least 2 of the following: sacroiliac joint tenderness and/or inflammatory lumbosacral pain; HLA-B27+; onset of arthritis in a male over 6 years of age; AAU; history of ankylosing spondylitis, ERA, sacroiliitis with inflammatory bowel disease, reactive arthritis, or AAU in a FDR	a,d,e
Undifferentiated	Arthritis that fulfills criteria in no category or in 2 or more of the above categories.	

Exclusion criteria: (a) Psoriasis or a history of psoriasis in the patient or FDR. (b) Arthritis in an HLA-B27-positive male beginning after the 6th birthday. (c) Ankylosing spondylitis, enthesitis-related arthritis, sacroiliitis with inflammatory bowel disease, reactive arthritis, acute anterior uveitis, or a history of one of these disorders in a FDR. (d) The presence of IgM rheumatoid factor on at least 2 occasions at least 3 months apart. (e) The presence of systemic JIA in the patient. Abbreviations: AAU, acute-anterior uveitis; ERA, enthesitis-related arthritis; FDR, first-degree relative; RF, rheumatoid factor. Table reproduced with permission from Stoll and Mellins^9^.

## The journey toward personalized treatment in JIA

An individual-patient-level understanding of disease pathophysiology and ideal treatment regimen continues to be a compelling goal of current research efforts. In JIA, both a patient’s underlying individual genetics and an understanding of the nature of a patient’s microbiome seem to play important roles in the development of JIA, progression of the disease, and response to treatment.

**Figure 1.**
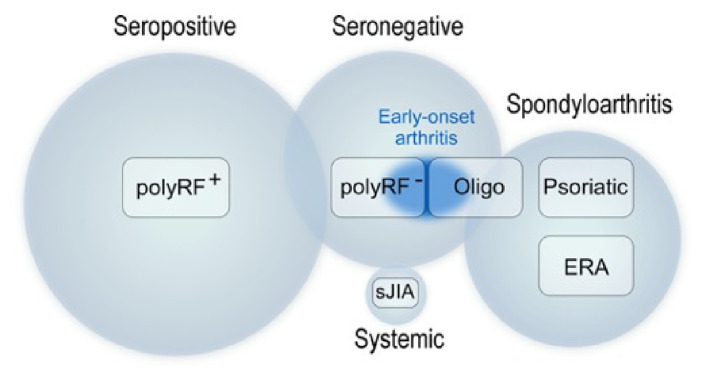
Proposed arthritis classification clusters based on current genetics studies. Boxes represent International League of Associations for Rheumatology (ILAR) categories of juvenile idiopathic arthritis (JIA). ERA, enthesitis-related arthritis; Oligo, oligoarticular JIA; polyRF+, polyarticular rheumatoid factor positive JIA; polyRF–, polyarticular rheumatoid factor negative JIA; sJIA, systemic JIA. Reused with permission from Nigrovic, Raychaudhuri, and Thompson^7^.

The genetic underpinnings of JIA and their relationships to the phenotypic categories of the disease is a key component to understanding the pathophysiology and classification of JIA. Recent studies have identified divergent underlying genetics and microRNA patterns among JIA, its various categories, and healthy controls^5,6^. In a commentary, Nigrovic *et al*. advocated for a JIA classification scheme rooted in causal genetics and proposed four main clusters: rheumatoid factor seropositive, seronegative (with a distinct group that begins in early childhood), spondyloarthritis, and systemic arthritis (see Figure 1)^5,7,8^. Classification of JIA categories continues to be a controversial topic^9^. Researchers also continue to gain a better genetic understanding of response to treatment in JIA patients. A recent study by Bašić *et al*. showed that polymorphisms in the catalase gene (responsible for decomposing hydrogen peroxide into water and oxygen) are associated with polyarticular JIA susceptibility and with response to tumor necrosis factor (TNF) inhibition therapy, suggesting an important role of oxidative stress in disease^10^. Knowing that leukemia patients can have altered responses to methotrexate based on *SLCO1B1* allele type, Ramsey and colleagues showed that certain alleles of this gene were associated with decreased response to methotrexate in JIA patients, indicating a likely need for increased dosages in patients with these alleles compared to non-carriers^11^. Poppenberg *et al*. used machine learning of transcriptomes from peripheral blood mononuclear cells to predict disease activity in patients with JIA, forecasting future use of this approach to assess response to therapy^12^. Further research regarding the genetics of disease susceptibility and treatment response will likely move the field towards both biology-based classification and personalized treatment approaches^13^.

In addition to genetics, environmental factors contribute to JIA pathogenesis. Research continues to pursue an understanding of the relationship between the gut microbiome and JIA onset, activity, and response to therapy. Early life events that influence the composition of the microbiota (method of delivery at birth, feeding during infancy, home environment, and exposure to antibiotics^14^) significantly affect the mucosal barrier, nutritional absorption, and immune system development and ultimately also influence the risk of developing JIA. Along these lines, JIA patients have been reported to have abnormal microbiota (dysbiosis) compared to healthy individuals^15^. Van Dijkhuizen and colleagues used a multicenter prospective observational cohort study to illustrate gut dysbiosis in JIA patients (particularly in relation to increased levels of *Erysipelotrichaceae* and *Faecalibacterium prausnitzii* and decreased levels of *Allobaculum*)^16,17^. However, relative abundance of individual pro- and anti-inflammatory strains of *F. prausnitzii* may be more relevant. A study by Grevich and colleagues suggested a link between oral dysbiosis and gingivitis and JIA, with JIA plaque microbiota analysis showing increased presence of *Haemophilus* and *Kingella* and decreased presence of *Corynebacterium*^18^. While studies continue to show correlations between dysbiosis, as well as decreased organismal diversity, and the development of JIA, it is still unclear if dysbiosis is simply a biomarker or if it actually contributes to the pathogenesis of JIA^19,20^. Nevertheless, JIA dysbiosis profiles seem to be unique and can be distinguished from healthy individuals and from other diseases using machine learning approaches^21,22^. Further study is needed to determine whether the microbiome is a useful therapeutic target in JIA and, if so, which approaches to alter the gut microbiome (fecal transplant, probiotics, or dietary alteration) are efficacious in JIA^15^.

Although an understanding of the underlying genetics and microbiome changes of an individual patient could be an important tool for informing treatment decision in the future, current practice does not take these into account when deciding optimal treatment courses for JIA patients.

## Treatment of JIA in the era of biologic modifiers

Biologic disease modifiers have revolutionized the treatment of JIA patients over the past quarter century, and JIA patients have better disease outcomes with modern treatments than do adult rheumatoid arthritis (RA) patients^23^. The data showing that currently approved biologic therapy for JIA is efficacious continue to accumulate, with improved outcomes and decreased rates of need for orthopedic surgery and of complications like uveitis in modern era patients compared to those from the pre-biologic era^24–26^. While TNF inhibition remains the mainstay of treatment, research continues to analyze and understand other targeted avenues, including interleukin-6 (IL-6) blockade, Janus kinase (JAK) pathway inhibition, T cell activation blockade, and anti-B cell therapy (see Table 2). Owing to clinical comfort and longer track record, most of these newer agents are still considered “second-line” behind TNF inhibitors, but data collection on their efficacy and safety continues.

**Table 2.** Biologic targeted treatment approaches to JIA.

Drug	Mechanism of action	FDA indications in children	EMA indications in children	Dosing	Adult approval (FDA & EMA)	Off label evidence (if not FDA or EMA approved in children with arthritis)
Adalimumab	TNF inhibition	pJIA (>4yo)	pJIA (≥2yo) ERA-JIA (≥6yo) PP (≥4yo) CD (≥6yo) NIU (≥2yo) HS (≥12yo)	SC every other week	RA PsA AS PP CD UC (EMA only) HS (EMA only) NIU (EMA only)	
Etanercept	TNF inhibition	pJIA (≥2yo) PP (≥4yo)	ERA-JIA (≥12yo) EO JIA (≥2yo) pJIA (≥2yo) PsJIA (≥12yo) PP (≥6yo)	1–2 times weekly SC	RA PsA AS PP	
Infliximab	TNF inhibition	UC (≥6yo) CD (≥6yo)	UC (≥6yo) CD (≥6yo)	IV every 6–8 weeks (after loading doses)	RA PsA AS PP UC CD	27,28
Golimumab	TNF inhibition	pJIA (≥2yo) PsJIA (≥2yo)	pJIA (≥2yo)	SC injection monthly or IV every 8 weeks (after loading doses)	RA PsA AS UC	
Certolizumab pegol	TNF inhibition	None	None	SC injections monthly (after loading doses)	RA PsA AS PP CD (FDA only)	
Rituximab	B cell depletion	None	None	2 IV infusions every 16–24 weeks	RA GPA MPA NHL CML PV	29–31,32
Anakinra	IL-1 blockade	CAPS	sJIA (≥8mo) CAPS (≥8mo) FMF (≥8mo)	SC daily	RA AOSD (EMA only)	33
Canakinumab	IL-1 blockade	sJIA (≥2yo) CAPS (≥4yo) TRAPS Hyper IgD FMF	sJIA (≥2yo) CAPS (≥2yo) TRAPS (≥2yo) Hyper IgD (≥2yo)	SC every 4 weeks	Gout (EMA only)	
			FMF (>2yo)			
Tocilizumab	IL-6 blockade	pJIA (≥2yo) sJIA (≥2yo) CRS (≥2yo)	pJIA (≥2yo) eoJIA (≥2yo) sJIA (≥1yo) CRS (≥2yo)	IV or SC every 4 weeks	RA GCA	
Sarilumab	IL-6 blockade	None	None	SC every 2 weeks	RA	
Secukinumab	IL-17 blockade	None	PP (≥2yo)	SC every 4 weeks (with or without loading dose)	PP PsA AS	34
Ixekizumab	IL-17 blockade	None	PP (≥6yo)	SC every 4 weeks (after loading dose)	PsA AS PP	
Brodalumab	IL-17 blockade	None	None	SC every 2 weeks (after loading dose)	PP	
Abatacept	T cell deactivation	pJIA (≥2yo)	pJIA (≥2yo)	IV every 4 weeks (after loading dose)	RA PsA	
Ustekinumab	IL-12 & IL-23 blockade	PP (≥12yo)	PP (≥6yo)	SC every 12 weeks (after loading, which can be SC or IV)	PsA PP CD UC	35,36

AOSD, adult-onset Still disease; AS, ankylosing spondylitis; CAPS, cryopyrin-associated periodic syndromes; CD, Crohn’s disease; CML, chronic myelogenous leukemia; EMA, European Medicines Agency; eoJIA, extended oligoarticular juvenile idiopathic arthritis; ERA-JIA, enthesitis-related arthritis juvenile idiopathic arthritis; FDA, Food and Drug Administration (United States); FMF, familial Mediterranean fever; GCA, giant cell arteritis GPA, granulomatosis with polyangiitis; HS, hidradenitis suppurativa; IL, interleukin; IV, intravenous; mo, months old; MPA, microscopic polyangiitis; NHL, non-Hodgkin lymphoma; NIU, non-infectious uveitis; pJIA, polyarticular juvenile idiopathic arthritis; PP, plaque psoriasis; PsA, psoriatic arthritis; PsJIA, psoriatic juvenile idiopathic arthritis; PV, pemphigus vulgaris; RA, rheumatoid arthritis; SC, subcutaneous; TNF, tumor necrosis factor; TRAPS, tumor necrosis factor receptor associated periodic syndrome; UC, ulcerative colitis; yo, years old.

### Treatment agents

TNF inhibitors are a reliable way to induce remission in non-systemic JIA patients and are the backbone of current JIA treatment regimens^37,38^. TNF inhibitors are divided into two classes: monoclonal antibodies against TNF (infliximab, adalimumab, certolizumab pegol, and golimumab) and a receptor fusion protein (etanercept). The monoclonal antibodies against TNF provide better treatment for granulomatous conditions, including inflammatory bowel disease and uveitis. There are several recent publications addressing the benefits of TNF inhibitors in treating JIA. TNF inhibitors are most effective when given early on in disease course^39^. Standard dosing regimens are occasionally adjusted based on clinical changes, but therapeutic drug monitoring is not yet a component of routine clinical management^40^. Immunogenicity (and thereby decreased efficacy) can occur with the use of TNF inhibitors and is diminished by concomitant methotrexate use in adult patients with RA; this has not been clearly shown in JIA, and clinical monitoring of anti-drug antibodies is not current standard of practice in pediatric rheumatology care unless decreased clinically efficacy is noticed^41–44^. A common concern with patients on TNF inhibitors revolves around the long-term effects of drug therapy, including infection risk, malignancy risk, and adverse drug effects. Large registry studies and systematic reviews continue to find that, overall, TNF inhibition is safe, effective, and well tolerated^45–51^.

Another targetable pro-inflammatory cytokine for children with arthritis is IL-6. Recent updates have been published detailing the role of IL-6 blockade in treating JIA. Tocilizumab is an antibody against the IL-6 receptor that can be used as an alternative treatment for JIA and is available in intravenous and subcutaneous forms. It can be particularly effective for previously treatment-resistant patients^52^ but has the potential adverse effects of cytopenias and elevated transaminases^47,53^ and similar infection rates to TNF inhibition^24^. Significant adverse events with tocilizumab are more likely to occur with concomitant immunosuppression^54^. Tocilizumab continues to be an option in the management of JIA, particularly in previously treatment-resistant patients, but regular monitoring for adverse effects is necessary^55^.

Beyond cytokine blockade, disruption of T cell co-stimulation is an alternative approach to treating JIA. Abatacept (CTLA-4-Ig) interferes with T cell activation and is a newer treatment option for patients with JIA. Abatacept is available in both intravenous and subcutaneous forms, providing options for optimal treatment plans for patients. Research continues to pursue an understanding of abatacept’s efficacy, adverse event profile, and overall effect on the immune system. Open label trials and systematic reviews consistently show a favorable safety profile^24,46,54,56^. Given abatacept’s effects on T cells, there has been concern over the implications of this therapy on vaccine-induced immunity; however, a substudy of 222 patients with polyarticular JIA receiving subcutaneous abatacept showed maintenance of effective diphtheria and tetanus vaccination protection^57^. Abatacept remains an option for biologic treatment in patients with JIA, including those with uveitis.

Clinicians and researchers continue to show interest in using other biologic modifiers for the treatment of JIA, particularly those approved for adult RA. Agents of interest include those blocking IL-1 (anakinra and canakinumab), IL-17 (secukinumab and ixekizumab), and IL-12/23 (ustekinumab) as well as Janus kinase inhibitors (tofacitinib, baricitinib, upadacitinib, and filgotinib). Tofacitinib has recently been approved by the FDA for polyarticular JIA and is available in an oral solution^58^. Rituximab, a monoclonal antibody targeting B cells, is not licensed specifically for JIA, but it can still be a treatment option for these patients, with an effective response and a low rate of adverse events^54,59–62^.

Patients treated with biologic modifiers have better disease outcomes than patients in the pre-biologic era, with lower rates of complications overall. The data showing that currently approved biologic therapy for JIA is efficacious continue to accumulate^24^. Berthold *et al*. collected JIA cases between 2002 and 2010 in part of Sweden and noted the need for orthopedic surgery and the presence of uveitis were diminished in comparison to studies of patients diagnosed over 20 years ago. However, active disease was still experienced by these patients more than 50% of the time, indicating persistent treatment challenges despite the development of biologic therapy^25^. Giancane *et al*. compared two groups of JIA patients, methotrexate era (disease presentation before 2000) versus biologic era (disease presentation after 2000), using a cross-sectional assessment to assess disease activity and joint damage and found significant improvement in outcomes in the biologic-era patients compared to the methotrexate-era patients^26^. Arnstad *et al*. used the Nordic cohort to show that early self-reported disease-related pain predicted persistent pain and unfavorable long-term disease outcomes^63^. Henrey *et al*. employed a fine-tuned Nordic model to predict non-achievement of remission and severe disease course in a cohort of Canadian JIA patients, illustrating the potential utility of this model in outcomes research^64^. TNF inhibitors are the backbone of biologic therapy with other available options, including abatacept and tocilizumab, while data continue to be collected on the use of other biologic agents for the treatment of JIA^27–33,35,64,65^. Biologic treatments have revolutionized care for JIA patients; however, there is still an ongoing need for new medications to treat JIA, particularly jaw arthritis^65,66^.

### Temporomandibular involvement

The temporomandibular joint (TMJ) continues to be a difficult-to-treat and common site of involvement for juvenile arthritis, affecting mandibular growth and development and causing potential deformity and adverse quality of life outcomes^67–70^. A Danish cohort study showed a cumulative incidence of orofacial symptoms in JIA approaching 50% at 5 years after JIA onset^71^. The ideal methods for identifying, imaging, and scoring TMJ dysfunction, as well as its treatment, continue to be topics of much discussion, but magnetic resonance imaging with contrast of the TMJ read by a radiologist with experience in TMJ arthritis imaging is the current standard of care^72–77^. Standardization of measurement of TMJ involvement has been developed by an international working group, and there is also an effort to identify the ideal timing of potential surgical intervention^78–81^. There is evidence to support the notion that treatment of arthritis with systemic therapy is also beneficial for the TMJ; however, optimal treatment of TMJ arthritis in children with JIA remains a significant challenge^82,83^.

### Treatment guidelines and decision-making

In 2019, the American College of Rheumatology (ACR) and Arthritis Foundation updated treatment guidelines for JIA from their previous 2011 guidelines, defining patient populations by clinical phenotypes rather than ILAR categories, in line with current understanding of biology and treatment response. Major changes included removal of NSAID monotherapy as first-line treatment for polyarthritis. In fact, in the presence of certain risk factors (joint damage or positive anti-cyclic citrullinated peptide antibodies), first-line treatment for polyarthritis can be biologic therapy. Given mostly equivalent safety and efficacy data between biologic agents (and overall lack of head-to-head comparison studies), specific biologic modifiers are only recommended in particular scenarios: TNF inhibitors are specifically recommended for sacroiliitis, and rituximab is recommended only after TNF inhibition, abatacept, and tocilizumab have been tried. The overall quantity and quality of evidence continues to be low, and shared decision making among clinicians, patients, and parents continues to be recommended. The guidelines make no recommendations regarding agents for JAK inhibition, IL-17 blockade, and IL-12/23 inhibition but point towards studies underway to help determine the future role for these therapies in JIA treatment^38^.

In addition to treating the arthritis associated with JIA, the ACR and Arthritis Foundation produced a 2019 guideline for screening, monitoring, and treating JIA-associated uveitis. The British Society for Pediatric and Adolescent Rheumatology, in coordination with the Royal College of Ophthalmologists, has also produced a similar guideline^84^. These guidelines were limited by lack of high-quality evidence and few randomized controlled trials, instead relying mainly upon expert opinion. Regular screening for uveitis is emphasized in JIA patients as well as regular monitoring of patients with previously diagnosed uveitis, particularly around times of transitions in therapy. Methotrexate, adalimumab, and infliximab are recommended when systemic treatment is needed for uveitis^85^, and a multicenter, double-blind, randomized, placebo-controlled trial has demonstrated efficacy of adalimumab with methotrexate compared to methotrexate alone in the treatment of JIA-associated uveitis^86^. In addition to anti-TNF monoclonal antibodies, tocilizumab, rituximab, and abatacept may all have roles in treating refractory uveitis in the setting of JIA.

Skillful JIA management relies upon accurate assessment of disease severity, possibility of achieving remission, likelihood of relapse, and overall treatment efficacy to inform strategic treatment decisions. Accurate assessment of clinical disease activity involves a combination of clinician assessment, patient assessment, and laboratory criteria. Widely used current clinical assessment criteria are not perfect tools, overlooking some aspects of disease and overemphasizing other factors^87^. Clinical, serologic, and imaging predictors of disease remission are still elusive, leaving clinical expertise and experience as the main compass for navigating treatment decisions prior to achievement of clinical remission^88–90^. Numerous models have been developed for the prediction of disease severity, risk of non-achievement of remission, and risk of relapse; however, JIA category continues to be the strongest predictor of remission, with highest rates of remission in persistent oligoarticular JIA and lowest rates of remission in rheumatoid factor-positive polyarticular JIA^62,63,91–97^. Utilization of these tools to drive treatment decisions is helpful, but patient involvement in shared decision making is also an important component in treatment^98^. Data from multiple registries suggest that a “window of opportunity” for early treatment with disease-modifying therapies is associated with better disease control and outcomes, underscoring the importance of early aggressive therapy at time of diagnosis^99^. With the availability of numerous effective therapies, the concept of treat-to-target (with target being remission or low disease activity) is an effective guide to decision making in the care of JIA patients^100–102^.

## Conclusion

Research continues to push JIA treatment towards the era of therapy personalization with better understandings of underlying genetics and the role of the microbiome. Treatment increasingly relies on biologic modifier therapy, and patient outcomes continue to improve with aggressive therapy. Uveitis and TMJ involvement continue to complicate JIA treatment decisions, and guidelines have yet to include less common biologic therapies for refractory cases. Disease category continues to be the best predictor for remission, but biomarker and polymorphism data hold promise as possibilities for more personalized approaches to JIA treatment in the future.

## Author contributions

John M. Bridges undertook investigation, visualization, and preparation of the original draft. Elizabeth D. Mellins and Randy Q. Cron undertook the conceptualization, supervision, and reviewing and editing of the manuscript.
